# The emerging roles of METTL1-mediated tRNA m^7^G methylation in cancer development and immunotherapy

**DOI:** 10.3389/fimmu.2025.1706984

**Published:** 2026-01-05

**Authors:** Qiang Wang, Xiulin Jiang, Yixiao Yuan, Chunhong Li

**Affiliations:** 1 Department of Gastrointestinal Surgical Unit, Suining Central Hospital, Suining, Sichuan, China; 2 Department of Systems Biology, City of Hope Comprehensive Cancer Center, Biomedical Research Center, Monrovia, CA, United States; 3 Department of Oncology, Suining Central Hospital, Suining, Sichuan, China

**Keywords:** METTL1, tRNA modifications, cancer progression, tumor microenvironment, tumor immunity, immunotherapy, epigenetic regulation, biomarker

## Abstract

RNA modifications, particularly N7-methylguanosine (m^7^G), have emerged as critical epigenetic regulators in cancer biology. METTL1, a conserved S-adenosylmethionine-dependent methyltransferase, catalyzes m^7^G modification primarily on tRNA, often in complex with its cofactor WDR4. This modification stabilizes tRNA structure, protects it from degradation, and enhances the translation efficiency of specific codons, thereby enabling selective protein synthesis. Aberrant METTL1 expression has been observed across multiple cancer types—including lung, liver, colorectal, gastric, breast, cholangiocarcinoma, esophageal, glioma, head and neck, and thyroid cancers-where it promotes tumor proliferation, metastasis, therapy resistance, and metabolic reprogramming. Mechanistically, METTL1-mediated tRNA m^7^G modification influences downstream mRNA stability and translation, affecting oncogenes, drug resistance genes, and key metabolic regulators. Moreover, METTL1 shapes the tumor immune microenvironment by modulating immune cell infiltration, promoting immunosuppressive populations, and contributing to immune evasion, which has implications for immunotherapy. Collectively, METTL1 functions as a pivotal driver of cancer progression and represents a promising biomarker and therapeutic target, highlighting the potential of targeting tRNA m^7^G modification in precision oncology.

## 1 Introduction

RNA modifications are increasingly recognized as a component of epigenetic regulation, participating in fine-tuning gene expression from post-transcriptional processing to translation (1). To date, dozens of chemical RNA modifications have been identified, including N6-methyladenosine (m^6^A), 5-methylcytosine (m^5^C), pseudouridine (Ψ), N1-methyladenosine (m^1^A), and m^7^G, which are distributed across mRNAs, tRNAs, rRNAs, long non-coding RNAs (lncRNAs), and small RNAs (2). Analogous to DNA methylation and histone modifications, RNA methylation can alter the physicochemical properties, conformation, and protein-binding capacity of RNA, thereby influencing splicing, nuclear export, stability, subcellular localization, and translation efficiency. Consequently, RNA modifications provide a reversible, dynamic, and highly responsive regulatory layer that enables cells to rapidly reprogram gene expression in response to environmental and physiological changes (2).

Aberrant RNA methylation has been implicated in a wide range of pathological conditions, with cancer research being particularly active. By modulating mRNA translation efficiency or altering non-coding RNA function, RNA modifications can promote or inhibit cell proliferation, apoptosis, metabolic reprogramming, and cell fate decisions (3). Furthermore, RNA modifications can influence immune recognition, for instance by affecting antigen peptide generation or intracellular signaling in immune cells, thereby playing critical roles in the tumor immune microenvironment (4). Collectively, understanding RNA methylation provides insight into the nuanced regulation of gene expression and offers opportunities to identify novel biomarkers and therapeutic targets.

m^7^G is a prevalent nucleoside methylation modification, present not only at the 5′ cap of mRNAs but also internally in tRNAs and rRNAs (5). In tRNAs, m^7^G modifications typically occur in the variable loop or proximal regions (commonly annotated as G46/47, depending on species and tRNA type) (6). The methyl group added at the N7 position of guanine alters base charge distribution and stacking properties, thereby affecting local conformation and overall tRNA folding stability. Functionally, m^7^G modifications in tRNAs are crucial for maintaining structural integrity, protecting against nuclease degradation, and optimizing translation efficiency (7). These modifications can influence the structure surrounding the anticodon or interactions with translation-related proteins, such as aminoacyl-tRNA synthetases or ribosomal factors, thereby enabling codon-specific translation regulation. Such tRNA modification–mediated translational control is considered a key mechanism by which cells rapidly adjust protein expression profiles under stress, differentiation, or pathological conditions.

METTL1 (Methyltransferase Like 1) has been identified as a conserved methyltransferase responsible for catalyzing m^7^G formation in tRNAs (8). METTL1 typically functions as a heterodimeric complex with its cofactor WDR4 (WD repeat domain 4), where METTL1 provides catalytic activity and WDR4 contributes to substrate recognition, structural stabilization, or localization (8). This complex is highly conserved across eukaryotes, indicating its fundamental biological role. Functional studies have demonstrated that METTL1-mediated tRNA m^7^G modification is essential for maintaining tRNA chemical and conformational stability, reducing tRNA degradation, and enhancing translation efficiency for specific codons, thereby selectively promoting translation of dependent mRNAs (7). Moreover, METTL1 participates in cell cycle regulation, stress responses (e.g., oxidative stress, heat shock), and developmental programs by reprogramming protein synthesis. Loss-of-function experiments (e.g., METTL1 knockout or knockdown) typically result in impaired tRNA homeostasis, reduced global or codon-specific protein synthesis, and decreased proliferation or increased stress sensitivity, supporting its role as a central regulator of translation (9). With the advent of high-throughput sequencing and refined RNA modification profiling techniques—such as selective immunoprecipitation, LC-MS/MS quantification, and chemical-based site mapping—the significance of METTL1 and m^7^G modifications in tumor biology has become increasingly evident. Emerging evidence indicates that METTL1 is aberrantly expressed in various cancers, where its upregulation is associated with enhanced tumor cell proliferation, migration, chemoresistance, and poor prognosis (10). Additionally, METTL1 may influence the tumor immune microenvironment by modulating antigen presentation or affecting immune effector cell function to facilitate immune evasion (11). Nonetheless, several gaps remain: the precise molecular mechanisms underlying METTL1’s selective regulation of specific mRNA translation are not fully elucidated; the functional heterogeneity of METTL1 across tissues and tumor types requires validation with larger clinical cohorts and multi-omics analyses; and the development of specific METTL1 inhibitors or clinically feasible interventions is still in its early stages.

This review aims to systematically summarize the biological functions of METTL1, its roles in tumor development and tumor immunity, as well as current therapeutic attempts and future research directions. By integrating molecular mechanisms and clinical relevance, we aim to provide readers with a comprehensive overview, highlight critical knowledge gaps, and offer theoretical and methodological guidance for the translational development of METTL1 as a biomarker or therapeutic target.

## 2 Molecular functions and biological roles of METTL1

### 2.1 METTL1-mediated tRNA m^7^G methylation

METTL1 (Methyltransferase Like 1) is a conserved S-adenosylmethionine (SAM)-dependent methyltransferase that catalyzes the m^7^G modification in RNA (11). The protein structure consists of several functional modules, among which the catalytic methyltransferase domain, located from the N-terminal to the middle region, adopts a typical Rossmann-like fold capable of binding SAM and transferring the methyl group to the N7 position of the target guanine via key active-site residues (12). In addition to the catalytic core, METTL1 contains an RNA-binding region enriched in positively charged residues, allowing specific recognition of RNA substrates and ensuring efficient modification. METTL1 typically forms a heterodimeric complex with WDR4, in which the interface is composed of specific α-helices and β-sheets (12). This interaction not only stabilizes METTL1’s structure but also enhances its catalytic activity and substrate specificity (13). Overall, METTL1 executes methylation via its catalytic domain, recognizes RNA substrates through its RNA-binding region, and relies on WDR4 for complex formation, thereby achieving efficient and precise m^7^G modifications that play critical roles in RNA metabolism and gene expression regulation (14). METTL1 is the primary tRNA m^7^G methyltransferase, and its catalytic activity depends on the stable interaction with WDR4. The METTL1-WDR4 complex introduces N7-methyl modifications at specific sites in various tRNAs, mainly within the variable loop region (e.g., G46) (14). The m^7^G modification is conserved not only in higher eukaryotic tRNAs but also in model organisms such as yeast and C. elegans, indicating its fundamental evolutionary importance. Notably, m^7^G modifications are selectively distributed among different tRNA types, providing a molecular basis for “codon-biased translation” and allowing cells to fine-tune protein synthesis under distinct physiological or pathological conditions (15).

### 2.2 Effects on RNA stability, translation efficiency, and protein synthesis

**Figure 1:**
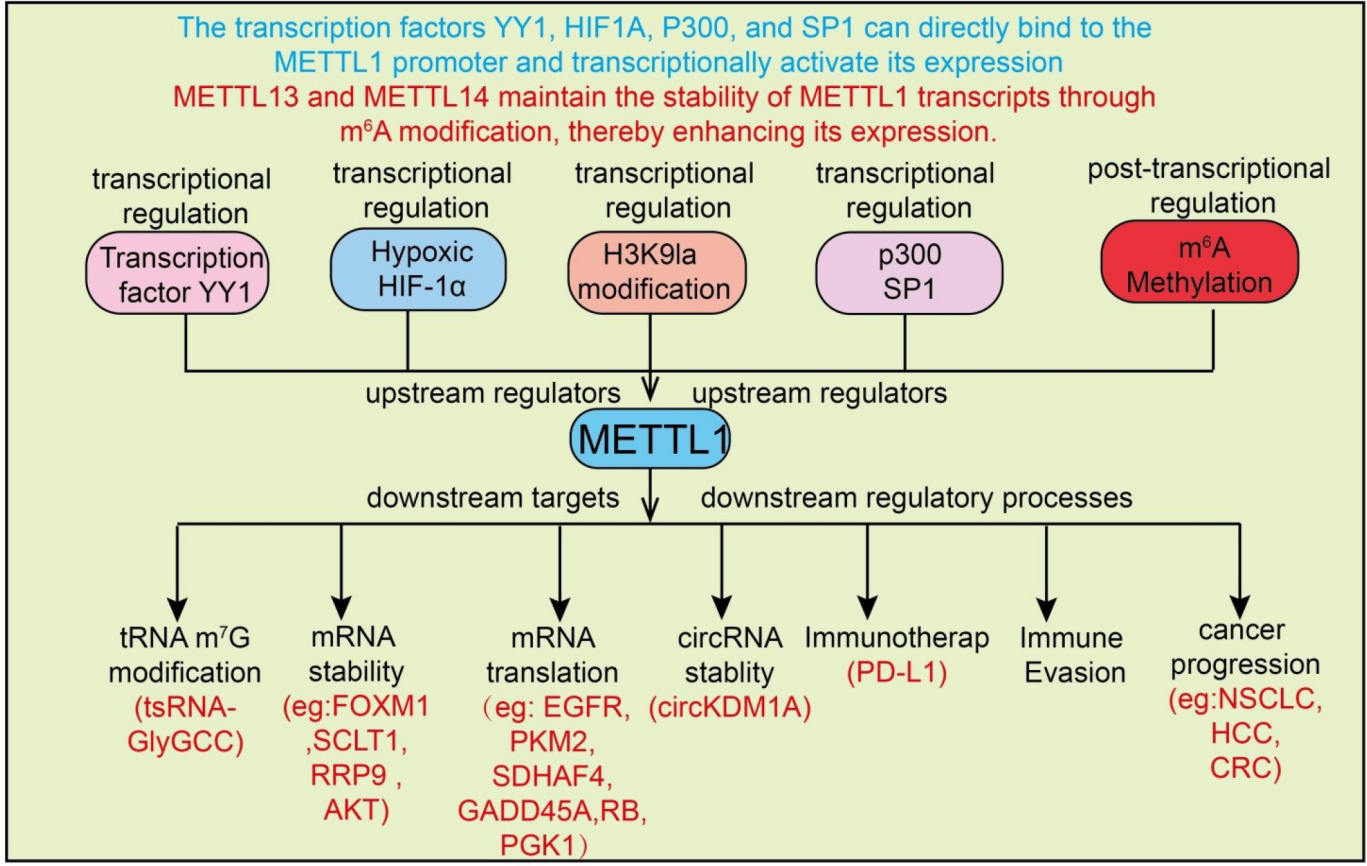
Upstream regulatory mechanisms of METTL1 and the downstream biological processes it participates in cancer. METTL1 expression is transcriptionally and post-transcriptionally regulated by multiple upstream factors. Transcription factors YY1, HIF1A, P300, and SP1 directly bind to the METTL1 promoter and activate its transcription. Post-transcriptionally, METTL13 and METTL14 maintain the stability of METTL1 transcripts through m^6A modification, enhancing its expression. METTL1 then regulates various downstream targets and processes, including tRNA m^7^G modification (e.g., tsRNA-GlyGCC), mRNA stability (e.g., FOXM1, SCLT1, RRP9, AKT), mRNA translation (e.g., EGFR, PKM2, SDHAF4, GADD45A, RB, PGK1), circRNA stability (e.g., circKDM1A), immunotherapy response (PD-L1), immune evasion, and cancer progression (e.g., NSCLC, HCC, CRC). Upstream regulators and downstream targets are indicated.

The m^7^G modification enhances tRNA structural stability and folding conformation, improving ribosomal accommodation and functional integrity (16). On one hand, m^7^G reduces the susceptibility of tRNAs to nuclease-mediated degradation, thereby prolonging tRNA half-life and maintaining the stability of the tRNA pool. On the other hand, m^7^G increases tRNA binding affinity to cognate aminoacyl-tRNA synthetases and ribosomal subunits, facilitating codon recognition and accelerating peptide elongation (14). Studies have shown that METTL1 deficiency markedly decreases the translation efficiency of mRNAs enriched with specific codons, thereby affecting downstream protein synthesis. This “selective translation” effect confers high specificity in regulating the cellular proteome, rather than causing a global enhancement or suppression of translation (17). Particularly under stress, differentiation, or disease conditions, METTL1-mediated translational reprogramming allows cells to rapidly adapt to environmental changes. METTL1-mediated m^7^G modification also enhances the stability of certain mRNAs (**Figure 1**). The expression of METTL1 in cancer is regulated by multiple upstream factors and epigenetic mechanisms. Transcription factors such as YY1 can directly activate or repress METTL1 transcription. Tumor microenvironmental conditions, including hypoxia, also influence METTL1 expression, often promoting its upregulation. Additionally, epigenetic modifications such as lactylation and m^6^A methylation further contribute to the dynamic regulation of METTL1 in cancer cells, highlighting its complex control and potential as a therapeutic target **(see****Figure 1**). The modification can protect mRNAs from exogenous nuclease degradation, increasing their half-life, and may influence RNA-binding protein (RBP) interactions to regulate mRNA decay pathways. In some cancer cells, METTL1 stabilizes mRNAs encoding pro-proliferative or anti-apoptotic genes, promoting tumor cell survival and growth (18). Beyond the 5′ cap structure, internal m^7^G modifications can also impact translation by improving ribosome recognition and initiation complex assembly. In cancer cells, METTL1-dependent m^7^G modifications are often enriched on oncogenes or signaling pathway-related mRNAs, supporting their efficient expression and facilitating proliferation, migration, and drug resistance (19).

### 2.3 Regulation of cell proliferation, apoptosis, and metabolic homeostasis

METTL1-mediated m^7^G modifications exert profound effects on cell fate. In terms of proliferation, m^7^G promotes efficient translation of proteins involved in cell cycle regulation, including cyclins and cyclin-dependent kinases, thereby accelerating mitotic entry (20); METTL1 loss often results in cell cycle arrest and reduced proliferation. Regarding apoptosis, METTL1 regulates the translation balance of pro- and anti-apoptotic factors, affecting cellular sensitivity to stressors such as DNA damage or oxidative stress; downregulation of METTL1 generally increases apoptosis susceptibility (8). METTL1 also contributes to metabolic homeostasis by selectively promoting the translation of enzymes and transporters involved in glucose metabolism, lipid biosynthesis, and amino acid balance, providing energy and biosynthetic precursors for rapidly proliferating tumor cells (21). This coupling with metabolic pathways partially explains why METTL1 is frequently upregulated in various cancers and is closely associated with tumor metabolic reprogramming.

## 3 Functional roles and mechanisms of METTL1 in cancer progression

**Figure 2:**
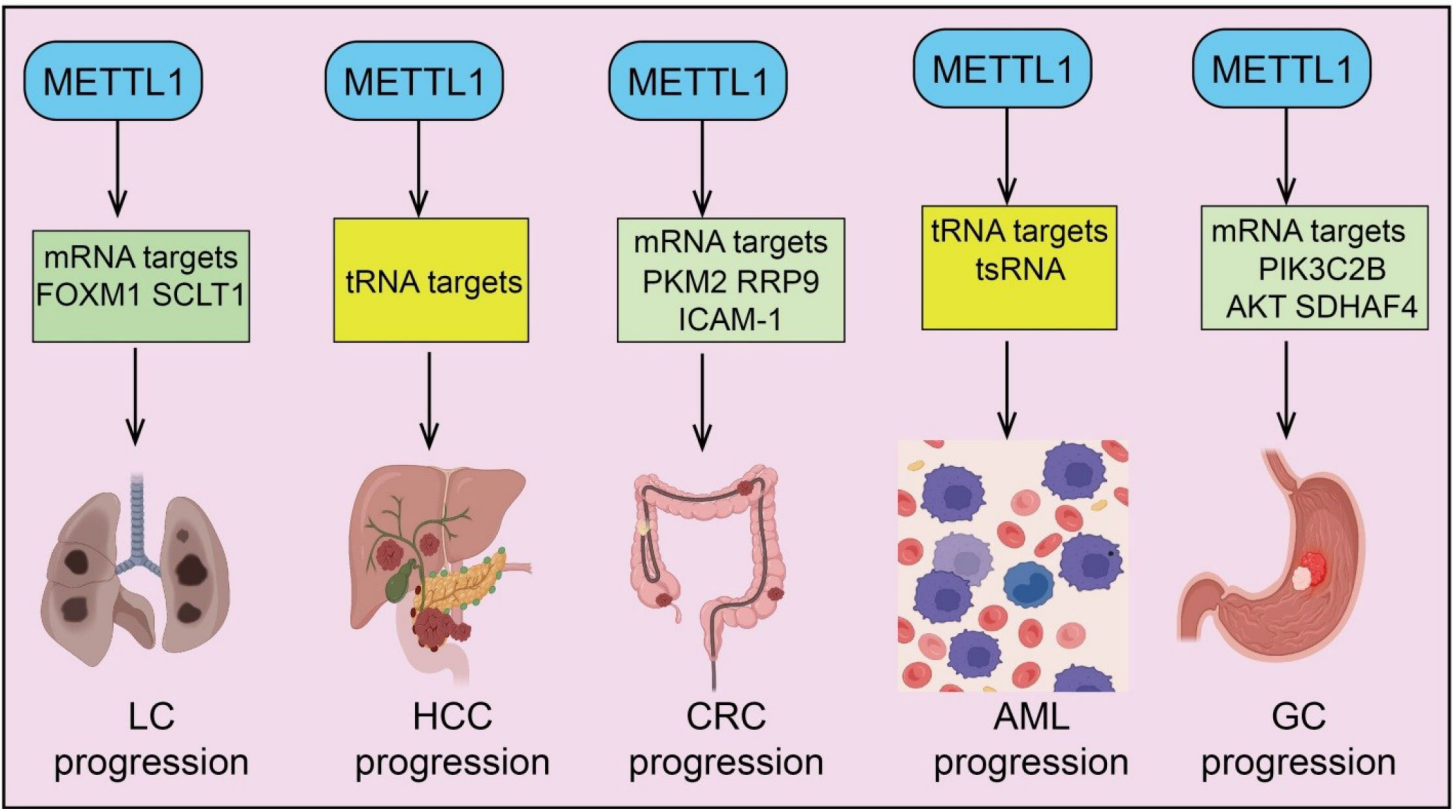
The mRNA and tRNA target genes of METTL1 in lung cancer, HCC, CRC, AML, and GC.

METTL1, an RNA m^7^G methyltransferase, plays a pivotal role in multiple cancers. It primarily mediates m^7^G modifications on tRNAs and mRNAs, thereby regulating target gene translation efficiency and mRNA stability, which in turn promotes tumor cell proliferation, migration, invasion, and drug resistance. Numerous studies have demonstrated that METTL1 is upregulated in a variety of malignancies, including lung cancer, hepatocellular carcinoma (HCC), colorectal cancer (CRC), acute myeloid leukemia (AML), gastric cancer, and breast cancer, and its high expression is often associated with poor patient prognosis (**Figure 2**).

METTL1 frequently functions in concert with its partner protein WDR4, and its expression can be modulated by upstream transcription factors or epigenetic modifications, forming a complex regulatory network. Mechanistically, METTL1-mediated tRNA m^7^G modifications enhance the translation of mRNAs enriched in codons decoded by these tRNAs, facilitating the selective synthesis of oncogenic proteins (22). Additionally, METTL1-mediated m^7^G modifications on mRNAs can increase transcript stability, further contributing to the accumulation of key proteins involved in tumor progression. Overall, METTL1 acts as a central regulator of cancer biology by integrating RNA modification-mediated translational control and mRNA stability, thereby driving tumor growth, metastasis, and therapy resistance. Its aberrant expression and multifaceted mechanisms make METTL1 a promising prognostic biomarker and potential therapeutic target in oncology.

### 3.1 Lung cancer

Aberrant RNA modifications have been increasingly recognized as critical contributors to lung cancer initiation and progression, with the m^7^G methyltransferase METTL1 emerging as a key regulatory factor (23). In lung cancer, components of the tRNA m^7^G methyltransferase complex, METTL1 and WDR4, are significantly upregulated and correlate with poor patient prognosis. Studies have demonstrated that loss of METTL1/WDR4 impairs tRNA m^7^G modifications, markedly inhibiting lung cancer cell proliferation, colony formation, and invasion, as well as reducing tumorigenicity *in vivo*. Gain-of-function and mutational analyses further confirmed that METTL1 promotes lung cancer growth and invasion through regulation of tRNA m^7^G modifications (18). Mechanistically, highly translated mRNAs are often enriched in codons decoded by m^7^G -modified tRNAs, and METTL1 knockdown reduces the translation efficiency of these mRNAs, highlighting the pivotal role of tRNA modifications and corresponding codon composition in translational control (18). In lung adenocarcinoma (LUAD), METTL1 is significantly upregulated, and its high expression is associated with poor prognosis. Mechanistic studies revealed that METTL1 enhances FOXM1 mRNA stability in an m^7^G -dependent manner, resulting in increased FOXM1 expression. Further analyses indicated that FOXM1 transcriptionally represses PTPN13 expression, thereby reducing LUAD cell sensitivity to gefitinib (24) (**Figure 2**). Consistent findings show that METTL1 overexpression in LUAD tissues promotes cell proliferation and colony formation while inhibiting autophagy via activation of the AKT/mTORC1 signaling pathway. METTL1 has also been implicated in resistance to EGFR tyrosine kinase inhibitors (EGFR-TKIs), a major clinical challenge in non-small cell lung cancer (NSCLC) despite durable complete responses in some patients (25). Notably, internal m^7^G modifications on mRNAs and the METTL1/WDR4 methyltransferase complex are significantly elevated in NSCLC specimens and correlate with EGFR-TKIs resistance. Functional experiments demonstrate that METTL1/WDR4 promotes gefitinib resistance through its mRNA internal m^7^G methyltransferase activity, validated both *in vitro* and in animal models. Integrative m^7^G MeRIP-seq and RNA-seq analyses identified SCLT1 (Sodium Channel and Clathrin Linker Protein 1) as a direct m^7^G modification target of METTL1/WDR4. Knockdown of METTL1/WDR4 reduces SCLT1 mRNA methylation and stability, whereas overexpression of wild-type METTL1, but not a catalytically inactive mutant, restores SCLT1 mRNA stability (26). Further investigation revealed that METTL1/WDR4-mediated m^7^G modification of SCLT1 promotes gefitinib resistance via activation of the NF-κB signaling pathway (26). These findings underscore the critical role of aberrant internal m^7^G mRNA modifications in EGFR-TKIs resistance and suggest that targeting the METTL1/WDR4–SCLT1–NF-κB axis may provide a potential therapeutic strategy to overcome drug resistance in NSCLC.

### 3.2 Hepatocellular carcinoma

Aberrant RNA modifications have been increasingly recognized as critical mechanisms driving HCC initiation and progression (27). As a tRNA m^7^G methyltransferase, METTL1 plays a pivotal role in regulating HCC cell proliferation and tumor development. Lenvatinib is a first-line therapy for advanced HCC; however, its efficacy is often limited by acquired drug resistance. Proteomic analyses have revealed that key components of the tRNA m^7^G methyltransferase complex, METTL1 and WDR4, are significantly upregulated in lenvatinib-resistant HCC cells. Functional experiments demonstrated that METTL1 knockdown suppresses HCC cell proliferation and promotes apoptosis under lenvatinib treatment, thereby overcoming resistance, whereas overexpression of wild-type METTL1 (but not catalytic-dead mutants) induces drug resistance (7). *In vivo* studies further confirmed that METTL1/WDR4-mediated tRNA m^7^G modification is crucial for promoting lenvatinib resistance, mechanistically by enhancing translation of genes associated with the EGFR signaling pathway. WDR4 forms a functional complex with METTL1 to regulate m^7^G modification of oncogenic mRNAs. WDR4 knockdown reduces both METTL1 mRNA and protein levels, indirectly affecting the formation of the WDR4–METTL1 complex. In HCC patient tissues, METTL1 and WDR4 expression levels are positively correlated, and their high expression is associated with advanced tumor stage and poor prognosis, suggesting a synergistic oncogenic role. Mechanistic studies indicate that METTL1-mediated tRNA m^7^G modifications preferentially enhance the translation of mRNAs enriched in codons decoded by these tRNAs, thereby promoting the expression of tumor-promoting proteins and driving HCC progression (28). Further *in vivo* validation using conditional Mettl1 knock-in and knockout mouse models combined with hydrodynamic transfection-based HCC models confirmed the essential physiological function of Mettl1 in liver tumorigenesis. Collectively, these findings elucidate a molecular mechanism whereby METTL1 promotes HCC development through tRNA m^7^G -dependent translational regulation, highlighting METTL1 as a potential therapeutic target in HCC (**Figure 2**).

### 3.3 CRC

CRC development is influenced by multilayered epigenetic regulation, among which RNA m^7^G modification and its regulator METTL1 play critical roles in promoting tumor progression and modulating the tumor immune microenvironment (29). CRC typically exhibits poor responses to immunotherapy, reflecting pronounced immune evasion features. Recent studies have demonstrated that METTL1-mediated m^7^G modification of PKM mRNA enhances PKM2 protein expression, thereby promoting tumor progression and glycolytic activity (19) (**Figure 2**). Mechanistically, PKM2 not only drives glycolysis but also facilitates H3K9la modification and activates METTL1 transcription, forming a positive feedback loop. Additionally, PKM2 dimerization and nuclear translocation upregulate CD155 expression, contributing to immune evasion in CRC (19). tRNA-derived small RNAs (tsRNAs), a class of non-coding RNAs generated from tRNA processing, have emerged as important regulators in cancer biology. In CRC tissues, 5′ half-tRNA 5′-tiRNA-Gly-GCC (tsRNA-GlyGCC) is upregulated and regulated by METTL1-mediated tRNA m^7^G modification. Both *in vitro* and *in vivo* experiments demonstrate that tsRNA-GlyGCC promotes tumor growth and mediates 5-fluorouracil (5-FU) resistance (30). Mechanistically, tsRNA-GlyGCC targets the transcription factor SPIB to regulate the JAK1/STAT6 signaling pathway, driving chemoresistance. Notably, co-delivery of 5-FU and tsRNA-GlyGCC inhibitors using synthetic poly(β-amino ester) nanoparticles effectively suppresses tumor growth and enhances CRC sensitivity to 5-FU without obvious toxicity, suggesting a potential therapeutic strategy to overcome drug resistance (30). Circular RNAs (circRNAs) have also been implicated in CRC progression, although their regulatory mechanisms remain incompletely understood. Several circRNAs are enriched with m^7^G modifications catalyzed by METTL1, primarily at GG motifs. Among them, circKDM1A is stabilized by METTL1-mediated m^7^G modification, preventing degradation (31). Functional assays confirm that circKDM1A promotes CRC cell proliferation, migration, and invasion, while mutation of the m^7^G sites significantly diminishes its oncogenic activity. Mechanistically, circKDM1A activates the AKT pathway via upregulation of PDK1, driving CRC progression (31) (**Figure 1**). Hypoxia and hypoxia-inducible factor 1α (HIF-1α) are key regulators of CRC progression and can modulate the epitranscriptome. Under hypoxic conditions, tRNA RNA modifications are more consistently altered than mRNA modifications, with tRNA m^7^G abundance significantly reduced. METTL1 mRNA expression is downregulated under hypoxia, and HIF-1α directly binds to the METTL1 promoter at HRE elements, repressing its transcription, revealing a mechanism by which the hypoxic microenvironment negatively regulates METTL1 and tRNA m^7^G modification in CRC. Further studies confirm that METTL1 is highly expressed in CRC tissues and cell lines. Knockdown of METTL1 significantly inhibits CRC cell proliferation, migration, invasion, and sphere formation, whereas overexpression promotes these phenotypes. METTL1 positively correlates with ribosomal RNA processing 9 (RRP9) and enhances RRP9 expression via m^7^G methylation of its mRNA, which activates the PI3K/AKT signaling pathway, promoting CRC growth, metastasis, and stemness (32). In HCT-116 xenograft models, METTL1 knockdown suppresses tumor growth, whereas RRP9 overexpression partially rescues this effect. In metastatic CRC, METTL1 expression is significantly upregulated. Mechanistic studies reveal that METTL1 stabilizes ICAM-1 mRNA via m^7^G modification, promoting tumor progression (33). RNA-seq analysis of METTL1-silenced CRC cells identifies ICAM-1 as a key downstream target whose mRNA stability depends on METTL1-mediated m^7^G modification. These findings highlight METTL1 as a crucial promoter of metastatic CRC and a potential therapeutic target (**Figure 2**).

### 3.4 AML

AML is a highly aggressive hematologic malignancy, with its development closely linked to epitranscriptomic regulation (34). Among these, METTL1-mediated tRNA m^7^G modification plays a critical role in controlling protein synthesis and leukemic cell proliferation. Studies have demonstrated that METTL1 and its partner WDR4 are significantly upregulated in AML patients and are associated with poor prognosis. *In vitro* experiments indicate that knockdown of METTL1 markedly inhibits AML cell proliferation and induces apoptosis (35). Mechanistic analyses reveal that METTL1 silencing significantly reduces m^7^G levels on tRNAs, thereby destabilizing tRNA molecules and promoting the generation of tRNA-derived small RNAs (tsRNAs) (**Figure 2**). Furthermore, nascent proteome profiling shows that either METTL1 knockdown or transfection of total tRNA derived from METTL1-deficient AML cells decreases overall translation efficiency in AML cell (35). These findings indicate that METTL1 promotes leukemic cell proliferation and protein synthesis by regulating tRNA m^7^G modification and stability, highlighting METTL1 as a potential therapeutic target for AML.

### 3.5 Gastric cancer

GC is one of the most prevalent human malignancies worldwide, yet its molecular mechanisms remain incompletely understood (36). Recent studies have identified Tetraspanin 31 (TSPAN31) as highly expressed in GC tissues, with elevated levels significantly associated with poor patient prognosis. *In vitro* experiments demonstrated that TSPAN31 regulates GC cell proliferation, migration, and apoptosis. Further analyses suggested a potential co-expression relationship among TSPAN31, METTL1, and CCT2, with all three genes showing positively correlated expression in GC (37). Functional assays revealed that downregulation of TSPAN31 could partially reverse the tumor-promoting effects of METTL1 and CCT2 overexpression. Additionally, both METTL1 and WDR4 are overexpressed in GC patients, with high expression significantly correlated with unfavorable prognosis (37). *In vitro* and *in vivo* experiments indicate that silencing the METTL1-WDR4 complex suppresses GC cell proliferation and migration, suggesting its oncogenic role in GC progression. Mechanistically, METTL1-WDR4 enhances m^7^G methylation of PIK3C2B and AKT mRNAs, increasing their stability and leading to p-AKT overexpression, thereby activating downstream signaling pathways that promote malignant behaviors (38) (**Figure 2**). The transcription factor YY1 not only promotes the transcription of METTL1 and WDR4 but is also positively regulated by METTL1-WDR4 through increased m^7^G modification, forming a positive feedback loop. Moreover, knockdown of METTL1 reduces m^7^G -modified tRNA levels, impairing translation of oncogenes enriched in oxidative phosphorylation pathways. METTL1 also promotes translation of succinate dehydrogenase assembly factor 4 (SDHAF4), enhancing mitochondrial electron transport chain complex II (ETC II) activity, thereby accelerating GC metabolism and progression (39). Forced expression of SDHAF4 or chemical modulation of ETC II reverses the oncogenic effect of METTL1 in murine GC models (39). Collectively, these findings suggest that the METTL1/WDR4-mediated tRNA m^7^G modification promotes GC progression through regulation of oncogene translation and mitochondrial metabolism, highlighting this complex and its downstream signaling axis as potential therapeutic targets.

### 3.6 Breast cancer

BRCA is a prevalent malignancy whose initiation and progression are regulated, in part, by RNA epigenetic modifications (40). Recent studies indicate that METTL1-mediated tRNA m^7^G modification plays a crucial role in breast cancer cell proliferation, cell cycle regulation, and therapeutic response. In breast cancer, tRNA m^7^G modifications are essential for tRNA functionality; however, the biological role of METTL1-mediated tRNA m^7^G in BRCA remained unclear. Research has shown that components of the tRNA m^7^G methyltransferase complex, METTL1 and WDR4, are downregulated at both mRNA and protein levels in breast cancer tissues. Functionally, METTL1 exerts its effects in an enzymatic activity-dependent manner, suppressing breast cancer cell proliferation and arresting cell cycle progression (20). Mechanistically, METTL1 increases m^7^G levels on 19 specific tRNAs, thereby regulating translation of GADD45A and RB1 in a codon-dependent manner, influencing cell cycle control. Further *in vivo* experiments demonstrated that METTL1 overexpression enhances the anti-tumor efficacy of the CDK4/6 inhibitor abemaciclib. Overall, these findings reveal a tumor-suppressive role of METTL1-mediated tRNA m^7^G modification in breast cancer, selectively promoting translation of GADD45A and RB1 to induce G2/M cell cycle arrest, suggesting a potential strategy to improve CDK4/6 inhibitor therapy (20). Bioinformatic analyses further indicate that METTL1 expression in invasive breast carcinoma is closely associated with disease diagnosis, prognosis, and tumor immune infiltration. Analysis of TCGA data revealed that METTL1 expression is significantly higher in BRCA tissues compared with normal tissues, showing good diagnostic potential (41). Survival analyses demonstrated that patients with low METTL1 expression exhibited superior five-year overall survival (OS) and disease-specific survival (DSS) relative to those with high expression, indicating that elevated METTL1 correlates with poor prognosis. Moreover, METTL1 and its associated core genes are positively correlated with immunosuppressive cell populations, including regulatory T cells (Tregs) and follicular helper T cells (Tfh), suggesting that METTL1 may contribute to shaping an immunosuppressive tumor microenvironment (41). These findings highlight METTL1 as a potential biomarker for prognosis and immune-targeted therapy in breast cancer.

### 3.7 Cholangiocarcinoma (ICC)

ICC is an aggressive malignancy with poor prognosis (42). Recent studies have revealed that METTL1-mediated tRNA m^7^G modification plays a critical role in the translational regulation of oncogenes and tumor progression in ICC. In ICC, cancer cells maintain survival and drive tumor progression by selectively enhancing the translation of specific oncogenic transcripts. tRNA m^7^G modifications and components of the methyltransferase complex, METTL1 and WDR4, are significantly upregulated in ICC and closely associated with poor patient prognosis. METTL1/WDR4 is essential for regulating ICC cell survival and proliferation (43). Mechanistically, METTL1-mediated m^7^G tRNA modification selectively promotes the translation of oncogenic mRNAs, particularly those involved in cell cycle regulation and the EGFR signaling pathway, by modulating the decoding efficiency of specific codons. Specifically, loss of METTL1 reduces the translational efficiency of mRNAs enriched in m^7^G -tRNA-decoded codons, leading to ribosome pausing and accumulation at these sites, which in turn suppresses global protein synthesis and activates eIF2α phosphorylation, thereby impairing translation initiation (43). Further evidence from overexpression and gene knockout mouse models demonstrates that METTL1-mediated tRNA m^7^G modification has a tumor-promoting role in ICC initiation and progression. These findings highlight the physiological function and molecular mechanism by which METTL1 regulates oncogenic mRNA translation and drives ICC progression, providing a theoretical basis for developing anticancer strategies targeting tRNA modifications.

### 3.8 Esophageal squamous cell carcinoma

ESCC is a highly malignant cancer with a tendency for recurrence. Recent studies indicate that METTL1 and its partner WDR4, which mediate tRNA m^7^G modification, play a crucial role in ESCC tumorigenesis, progression, and oncogene translation regulation (44). In ESCC, dysregulated RNA modifications facilitate the processing and translation of oncogenic mRNAs, thereby promoting tumor progression. The components of the tRNA m^7^G methyltransferase complex, METTL1 and WDR4, are significantly upregulated in ESCC tissues and correlate with poor patient prognosis. Functional assays demonstrate that METTL1 and WDR4, dependent on their tRNA m^7^G methyltransferase activity, promote ESCC progression both *in vitro* and *in vivo (*45). Mechanistically, knockdown of METTL1 or WDR4 reduces tRNA m^7^G levels, thereby decreasing the translation efficiency of oncogenic transcripts associated with the RPTOR/ULK1/autophagy pathway. Furthermore, conditional knockout and overexpression mouse models of Mettl1 confirm its critical role in ESCC tumorigenesis (45). These findings highlight the oncogenic function of aberrant tRNA m^7^G modification and suggest that METTL1 and its downstream signaling axis may serve as potential therapeutic targets in ESCC.

### 3.9 Glioma

Gliomas are common malignant brain tumors characterized by high invasiveness and poor prognosis (46). Recent studies indicate that METTL1-mediated m^7^G modification plays a critical role in promoting glioma cell proliferation, glycolysis, and tumor progression. Bioinformatic analyses reveal that METTL1 expression increases with tumor grade and is significantly higher in glioma tissues compared to adjacent non-tumor tissues (47). High METTL1 expression promotes glioma cell proliferation and is associated with common clinical risk factors. Survival analysis demonstrates that elevated METTL1 correlates with poor patient prognosis, and both univariate and multivariate Cox regression analyses suggest that METTL1 serves as an independent prognostic risk factor in glioma (48). Functional enrichment and pathway analyses further indicate that the oncogenic role of METTL1 in glioma may be related to the MAPK signaling pathway. Experimental studies confirm that METTL1 is upregulated in glioma tissues and cell lines. Functional assays show that METTL1 knockdown inhibits glioma cell proliferation and glycolysis and slows tumor growth in mouse models. Mechanistically, METTL1 promotes internal m^7^G modification of PGK1 mRNA, prolonging its half-life and enhancing its expression. Overexpression of PGK1 partially rescues the proliferation and glycolysis inhibition caused by METTL1 knockdown. These findings suggest that METTL1 drives glioma progression through post-transcriptional regulation of PGK1, highlighting its potential as a therapeutic target and prognostic biomarker in glioma (48).

### 3.10 Head and neck squamous cell carcinoma

HNSC is a prevalent malignancy whose progression and metastasis are regulated by complex molecular mechanisms (49). Recent studies indicate that METTL1-mediated tRNA m^7^G modification plays a crucial role in regulating HNSC tumor growth, signaling pathway activation, and the tumor immune microenvironment. In HNSC, cancer cells selectively enhance the translation of oncogenic transcripts to promote tumor progression. The m^7^G methyltransferase complex components METTL1 and WDR4 are upregulated in HNSC and correlate with poor patient prognosis. Functional experiments demonstrate that METTL1/WDR4 promotes HNSC progression and metastasis (50). METTL1 knockout reduces m^7^G levels in 16 tRNA species, thereby suppressing the translation of a subset of oncogenes, including those involved in the PI3K/AKT/mTOR signaling pathway. Notably, chemical modulation of the PI3K/AKT/mTOR pathway can reverse the oncogenic effects of METTL1. Single-cell sequencing further reveals that METTL1 deletion alters the tumor immune microenvironment and cell–cell interactions between tumor and stromal compartments in mouse models. Overall, METTL1 promotes HNSC malignancy by regulating global mRNA translation, including PI3K/AKT/mTOR pathway transcripts, while reshaping the tumor immune microenvironment, highlighting its potential as a therapeutic target. In OSCC, METTL1 is markedly upregulated and associated with poor prognosis (51). METTL1 knockdown inhibits OSCC cell proliferation and induces G1-phase cell cycle arrest. Mechanistically, METTL1 catalyzes m^7^G modification in the 5′ untranslated region (5′UTR) of NEK1 mRNA, enhancing NEK1 mRNA stability and positively regulating its expression (52). NEK1 silencing similarly suppresses OSCC cell proliferation and clonogenic capacity while inducing G1 arrest. Further studies show that in HNSC, METTL1 enhances TXNDC12 mRNA stability via m^7^G dependent mechanisms, leading to upregulated TXNDC12 expression. High TXNDC12 levels promote c-Myc interaction with USP5, inhibit proteasomal degradation of c-Myc, and activate the c-Myc signaling pathway, thereby facilitating tumor invasion, proliferation, and cisplatin resistance (53). Additionally, METTL1 expression in HNSC and gastrointestinal tumors is positively regulated by the m^6^A methyltransferase METTL3. METTL3-mediated m^6^A modification enhances METTL1 expression, which in turn binds to CDK4 mRNA and modulates its m^7^G modification, increasing CDK4 stability and promoting cell cycle progression. Functional assays demonstrate that METTL1 overexpression enhances proliferation in HNSC, ESCA, STAD, and COAD cells, while METTL1 knockdown-induced proliferation inhibition can be partially rescued by CDK4 overexpression (50). These findings reveal a coordinated role of m^6^A and m^7^G modifications in tumorigenesis and suggest the METTL1/CDK4 axis as a potential therapeutic target in digestive system cancers.

### 3.11 Papillary thyroid carcinoma

PTC is a common malignancy of the endocrine system, whose initiation and progression are regulated by multiple molecular mechanisms (54). Recent studies indicate that Recent studies have revealed that METTL1-mediated tRNA m^7^G modification plays a significant oncogenic role in the initiation and progression of PTC. Compared with normal thyroid tissues, METTL1 is markedly upregulated in PTC samples, and its high expression is closely associated with poor clinical outcomes. Functionally, METTL1 promotes the proliferation and metastasis of PTC cells in a manner dependent on its tRNA methyltransferase activity. Knockdown of METTL1 markedly reduces PTC cell proliferation and migration, indicating that its tumor-promoting effects rely on the presence of functional m^7^G tRNA modification (55). Mechanistically, this effect is driven by a codon-biased translation regulation mechanism mediated by m^7^G -modified tRNAs. Loss of METTL1 leads to a reduced abundance of specific m^7^G -modified tRNAs that decode certain codons enriched in tumor-related mRNAs (55). In PTC, TNF-α mRNA contains a high frequency of codons that rely on m^7^G-modified tRNAs for efficient translation. Consequently, METTL1 depletion impairs the translation efficiency of TNF-α, leading to diminished production of this cytokine (55). TNF-α acts as both a proinflammatory and pro-tumorigenic factor, promoting PTC cell proliferation, metastasis, and immune evasion through the activation of downstream pathways such as NF-κB signaling. Importantly, exogenous supplementation of TNF-α partially rescues the proliferative and metastatic deficiencies caused by METTL1 knockdown, functionally validating the existence of a “tRNA modification–codon recognition–translation regulation” axis in PTC progression (55). Furthermore, tissue microarray analysis confirmed a significant positive correlation among METTL1, its cofactor WDR4, and TNF-α expression levels in PTC tissues, highlighting their cooperative role in driving tumor aggressiveness (55). Collectively, these findings define a novel translational control mechanism through which METTL1-mediated m^7^G tRNA modification enhances codon-specific translation of oncogenic and immune-related mRNAs, thereby promoting PTC development and progression.

## 4 METTL1 and tumor immunity

In recent years, increasing evidence has highlighted the critical role of the m^7^Gmethyltransferase METTL1 in various cancers, particularly in modulating the tumor immune microenvironment, influencing immune evasion, and regulating sensitivity to immunotherapy (17). METTL1 not only regulates protein translation and metabolic reprogramming through tRNA m^7^G methylation but also selectively promotes the expression of key factors and chemokines, thereby facilitating the accumulation of immunosuppressive cells, such as polymorphonuclear myeloid-derived suppressor cells (PMN-MDSCs), regulatory T cells (Tregs), and follicular helper T cells (Tfh), ultimately shaping an immunosuppressive microenvironment (17). In HCC, CRC, BRCA, prostate cancer, and ICC, high METTL1 expression is consistently associated with immunosuppressive tumor microenvironments, immune evasion, and poor prognosis (56). Furthermore, METTL1 can directly influence tumor responsiveness to immunotherapy by regulating key immune checkpoints (e.g., PD-L1, CTLA-4), immune regulatory molecules (modulating interferon signaling), and proinflammatory immune cell activity, indicating its potential as a therapeutic target. Collectively, these findings suggest that METTL1 exerts multifaceted and multilayered effects on tumorigenesis and immune regulation, providing a foundation for understanding the tumor immune microenvironment and developing METTL1-based immunotherapeutic strategies.

### 4.1 Potential impact on immune cells

**Figure 3:**
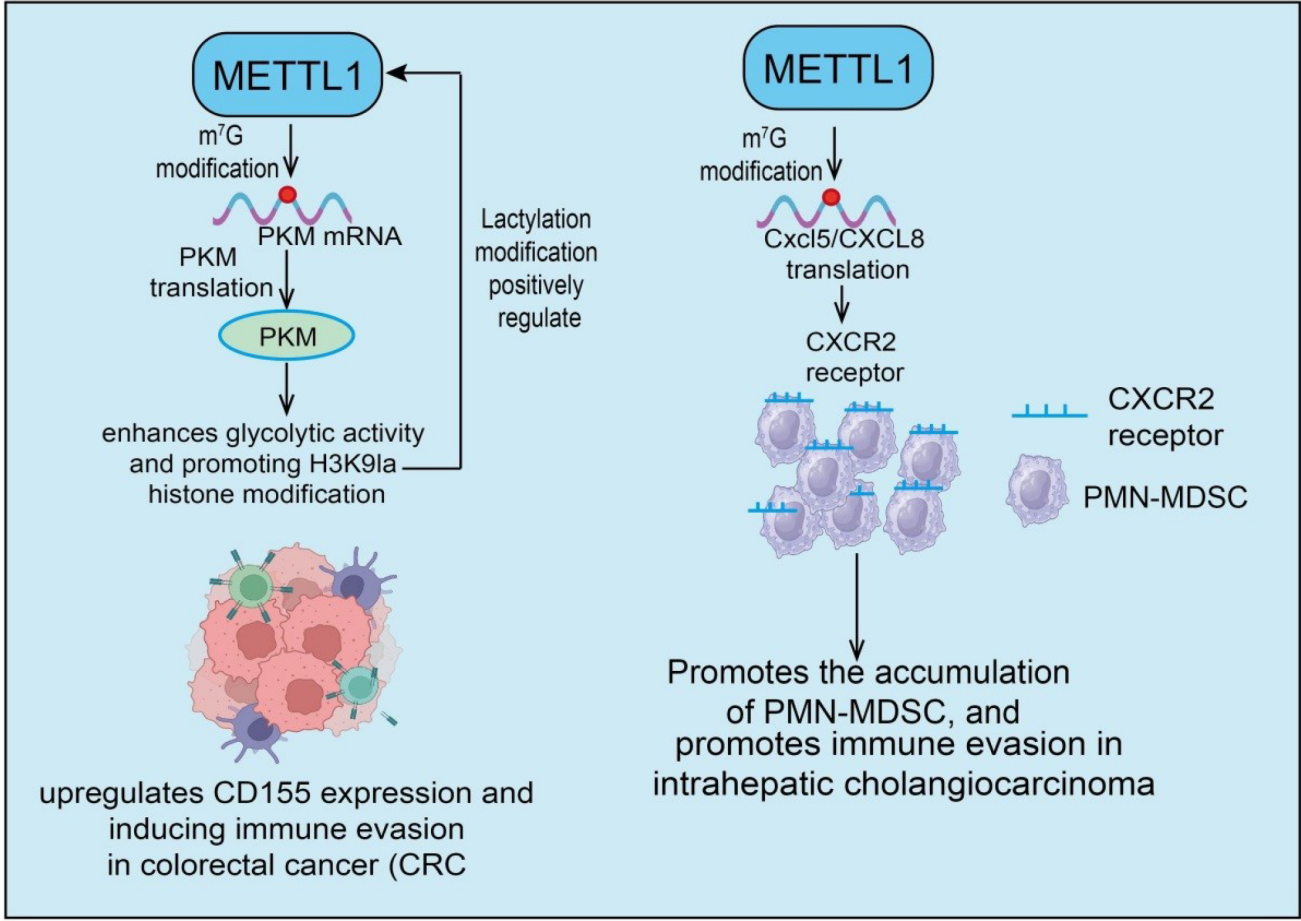
Functions and mechanisms of METTL1 in tumor immune cells and immune checkpoint regulation. Left panel: METTL1 catalyzes m^7^G modification on PKM mRNA, enhancing its translation. Elevated PKM promotes glycolytic activity and H3K9la histone modification, which further regulates METTL1 expression via a positive feedback loop. METTL1 also upregulates CD155 expression, inducing immune evasion in CRC. Right panel: METTL1-mediated m^7^G modification enhances translation of Cxcl5/CXCL8 mRNAs, activating the CXCR2 receptor on PMN-MDSCs, this promotes accumulation of PMN-MDSCs and facilitates immune evasion in intrahepatic cholangiocarcinoma.

Immune cells within the tumor microenvironment (TME) are critical determinants of tumor progression and therapeutic response. Various immune cell types—including T cells, B cells, natural killer (NK) cells, dendritic cells, and macrophages—play pivotal roles in recognizing and eliminating tumor cells (57). Tumor cells, however, exploit multiple immune evasion mechanisms to suppress immune cell activity, thereby promoting their own growth and metastasis (57). Recent studies indicate that epigenetic regulation and RNA modifications, such as METTL1-mediated m^7^G methylation, can modulate the expression of immune-related genes and the function of immune cells, offering new insights into the complex regulation of the TME.In HCC, the tumor cell-intrinsic roles of METTL1 in regulating PMN-MDSCs have recently been elucidated. METTL1, as a tRNA m^7^G methyltransferase, is upregulated in recurrent HCC following incomplete RFA (iRFA), accompanied by an increase in CD11b^+^CD15^+^ PMN-MDSCs and a decrease in CD8^+^ T cells (9). Mechanistically, heat stress induces METTL1 upregulation, enhancing TGF-β2 translation and promoting MDSC-mediated immunosuppression. Liver-specific METTL1 overexpression or knockdown significantly alters PMN-MDSC accumulation and modulates CD8^+^ T cell infiltration. Complete RFA effectively eliminates tumors, whereas iRFA-treated animals exhibit enhanced tumor growth and metastasis, with increased PMN-MDSCs and decreased CD8^+^ T cells (9) **(see****Figure 3**). Blocking the METTL1–TGF-β2–PMN-MDSC axis (e.g., via anti-Ly6G antibody, hepatocyte-specific METTL1 or Tgfb2 knockdown, or inhibition of TGF-β signaling) markedly attenuates iRFA-induced tumor progression and restores CD8^+^ T cell levels (9). These findings indicate that METTL1 promotes MDSC accumulation and an immunosuppressive microenvironment by regulating TGF-β2 translation, thereby impairing CD8^+^ T cell-mediated anti-tumor immunity—a key mechanism underlying HCC recurrence and immune evasion (9). However, this aspect has not been addressed in the manuscript, and to date, no studies have reported the impact of METTL1 deletion in T cells on the interferon pathway. In HCC, METTL1 upregulation drives nucleotide metabolic reprogramming, significantly affecting the immune microenvironment (58). METTL1-mediated metabolic reprogramming regulates the expression of key immune checkpoints, including PD-L1 and CTLA-4, and is associated with an immunosuppressive TME, reduced infiltration of activated T cells, and adverse clinical outcomes (58) In breast cancer, METTL1 expression is significantly elevated in tumor tissues compared to normal tissues, demonstrating diagnostic value. Patients with low METTL1 expression exhibit superior 5-year OS and DSS compared to high-expression patients, suggesting a link between METTL1 upregulation and poor prognosis. Further analysis reveals that METTL1 and its core associated genes are positively correlated with immunosuppressive cell types, including Tregs and Tfh cells, indicating a role in shaping an immunosuppressive TME. Integrative prognostic models incorporating METTL1 expression and immune checkpoint features show robust predictive performance in independent cohorts, highlighting potential clinical applications. In ACC, m^7^G modification and its regulatory genes significantly influence tumor prognosis, immune microenvironment, therapeutic response, and malignant progression (59). Lasso regression analysis identified a novel m^7^G risk signature (METTL1, NCBP1, NUDT1, and NUDT5), which provides high prognostic value, enhances traditional predictive models, and was validated in the GSE19750 cohort. Immune analyses demonstrate that a high m^7^G risk score correlates with increased glycolytic activity and suppression of anti-tumor immunity (59). Therapeutic correlation analysis suggests that the m^7^G risk score may predict responses to immune checkpoint blockade (ICBs) and mitotane treatment. Functional studies show that METTL1 promotes proliferation, migration, and invasion in ACC cells (60). Clinically, ACC samples with high METTL1 expression exhibit reduced CD8^+^ T cell infiltration and increased macrophage infiltration; METTL1 knockdown significantly inhibits xenograft tumor growth in mice (60). Mechanistically, METTL1 positively regulates the glycolytic rate-limiting enzyme HK1 and may be upstream regulated by miR-885-5p and the transcription factor CEBPB. Overall, METTL1 functions as a core m^7^G regulator, playing a pivotal role in ACC malignancy, immune modulation, and therapeutic responsiveness (60).

### 4.2 Tumor immune evasion

Tumor immune evasion is a critical mechanism driving cancer initiation, progression, and therapy resistance. Although the host immune system can recognize and eliminate abnormal cells, tumor cells effectively evade immune surveillance through multiple strategies, including modulating antigen expression, secreting immunosuppressive factors, upregulating immune checkpoints, and remodeling the TME (61). This immune evasion not only accelerates disease progression but also significantly impacts the efficacy of immunotherapies, representing a major focus in contemporary cancer research and treatment strategies. CRC typically exhibits poor responsiveness to immunotherapy and displays pronounced immune evasion features. RNA m^7^G modification plays a pivotal role in tumor development, and METTL1 has been shown to mediate m^7^G modification of PKM2 mRNA, enhancing its translation and upregulating PKM2 protein levels, thereby promoting tumor progression and glycolytic activity (19). Mechanistically, METTL1-catalyzed m^7^G methylation of PKM2 mRNA increases PKM2 protein expression, which not only augments glycolysis but also promotes H3K9la histone modification, activating METTL1 transcription and establishing a positive feedback loop. Further studies reveal that elevated PKM2 dimer formation and nuclear translocation upregulate CD155 expression (19), thereby inducing immune evasion in CRC (**Figure 3**). These findings indicate that METTL1 regulates a metabolism–epigenetic–immune axis, promoting tumor proliferation and metabolic reprogramming while modulating CD155-mediated immune escape, suggesting a potential intervention target for CRC immunotherapy. In HCC, integrated analyses combining single-cell sequencing and immune checkpoint-related signatures demonstrate that the METTL1/WDR4 complex and its associated RNA-based risk features are closely linked to immune evasion.

### 4.3 Role of METTL1 in tumor immunotherapy

Tumor immunotherapy has emerged as a major breakthrough in anticancer strategies, aiming to activate or enhance the host immune system to recognize and eliminate malignant cells, thereby improving patient outcomes (62). Unlike conventional surgery, radiotherapy, or chemotherapy, immunotherapy can exert sustained antitumor effects by targeting tumor-specific antigens. It encompasses diverse modalities, including ICIs, cellular therapies, cancer vaccines, and antibody-based therapeutics. Despite notable successes in certain cancers, tumor immune evasion and the immunosuppressive microenvironment limit the broad applicability of immunotherapy, driving research to explore novel mechanisms and potential targets that modulate antitumor immunity (63). Pan-cancer analyses have indicated that METTL1 expression is not significantly associated with patient sex, age, tumor stage, or treatment outcome; however, high METTL1 expression correlates with patient survival and may serve as a prognostic indicator (59). Functional enrichment analyses reveal that elevated METTL1 is significantly associated with tumor progression–related pathways. Furthermore, METTL1 expression demonstrates unique correlations with tumor immune infiltration and cancer stemness indices. In anti-PD-L1 therapy cohorts, responders exhibit significantly higher METTL1 expression than non-responders or partial responders, suggesting a potential link between METTL1 levels and immunotherapy sensitivity. Additional analyses indicate that tumor cell lines with high METTL1 expression are more sensitive to drugs targeting chromatin-associated histone methylation, ERK-MAPK, and WNT signaling pathways (59). These findings suggest that METTL1 not only regulates tumor biology but may also influence immune infiltration and stemness features, representing a potential biomarker for predicting immunotherapy response and drug sensitivity. Mechanistically, in primary and advanced prostate tumors, METTL1 is highly expressed, and its loss leads to tRNA m^7^G hypomethylation and the generation of novel 5′ tRNA-derived fragments (5′ tRFs). These 5′ tRFs selectively regulate translation, enhancing the synthesis of key tumor suppressors, interferon pathway components, and immune effector molecules, thereby modulating tumor cell functions at the molecular level (10). In prostate cancer models, METTL1 knockdown significantly increases the infiltration of proinflammatory immune cells within tumors and enhances immunotherapy efficacy (10). This demonstrates that METTL1-mediated tRNA m^7^G methylation is critical for translational control and tumor immune regulation, highlighting its potential as a therapeutically targetable molecule. In ICC, which exhibits extremely low response rates to ICIs, PMN-MDSCs are significantly enriched in advanced tumors and show strong positive correlations with METTL1 expression. Multiple *in vivo* cancer models reveal that METTL1 plays a central immunoregulatory role by controlling PMN-MDSC accumulation in the tumor microenvironment, thereby promoting ICC progression (64). Mechanistically, METTL1 selectively enhances the translation of human CXCL8 and murine Cxcl5, facilitating PMN-MDSC recruitment and tumor growth. Combined blockade of METTL1 and its downstream chemokine signaling markedly improves anti-PD-1 therapy efficacy in ICC models. These findings indicate that METTL1 regulates the accumulation of immunosuppressive cells via chemokine translation (64), representing a key mechanism underlying immune evasion and therapeutic resistance in ICC, and providing a potential target to enhance ICI efficacy.

In the current literature, studies investigating the role of METTL1 in tumor immunotherapy remain limited. To date, only a few reports have examined the potential functions of METTL1 in immunotherapy for prostate cancer and ICC, while systematic pan-cancer analyses-such as evaluating the correlation between METTL1 expression and ICI efficacy using TCGA datasets-are lacking. Recent data suggest that METTL1 expression is closely associated with features of the TME and may have potential predictive value for response to PD-1/L1 blockade in certain cohorts (59). For example, in the IMvigor210 anti-PD-L1 treatment cohort, patients with favorable responses exhibited significantly higher METTL1 expression compared to non- or limited-response groups, and high METTL1 expression was generally associated with a higher tumor neoantigen burden (59). However, in two anti–PD-1 cohorts, no significant differences in METTL1 expression were observed between responders and non-responders, indicating that its predictive value may be cancer type–specific (59). In addition, METTL1 expression was significantly correlated with stemness indices in multiple tumors, excluding a few cancer types, further suggesting a potential role in regulating tumor stemness and immune-related features (59). Collectively, these observations indicate that METTL1 may influence immunotherapy responses in certain cancers, but its pan-cancer relevance and underlying mechanisms require further investigation to inform individualized immunotherapy strategies.

## 5 METTL1 as a potential therapeutic target: advantages and challenges

As the role of METTL1 in cancer initiation and progression becomes increasingly evident, its potential as a therapeutic target has attracted growing attention. Currently, direct small-molecule inhibitors of METTL1 remain in the early stages of exploration, with limited reports primarily focused on *in vitro* activity validation and pharmacodynamic evaluation. Structural studies indicate that METTL1 possesses a canonical methyltransferase domain and utilizes S-adenosylmethionine (SAM) as a key cofactor, providing a theoretical basis for the design of SAM-competitive inhibitors (11). Meanwhile, RNA interference (RNAi) and CRISPR/Cas9 gene-editing technologies have been widely employed to validate METTL1 function, demonstrating that silencing or knockout of METTL1 can significantly suppress tumor cell proliferation and migration. These molecular tools provide an experimental foundation for the future development of RNA-based therapeutics, such as siRNAs, shRNAs, or antisense oligonucleotides (ASOs).

Recent studies further indicate that METTL1 not only affects intrinsic tumor cell biology but also modulates the tumor immune microenvironment. For instance, METTL1-mediated translational reprogramming can alter antigen processing and presentation in tumor cells, thereby influencing immune recognition (10). Moreover, its potential regulatory effects on tumor-associated immune cells—including macrophages, dendritic cells, and T lymphocytes—are receiving increasing attention. These findings suggest that targeting METTL1 could enhance tumor sensitivity to ICIs and help overcome certain forms of immunotherapy resistance.

In some cancers (e.g., ICC, hepatocellular carcinoma), combining METTL1 inhibitors with ICIs may have synergistic effects (58). However, due to the complex heterogeneity of tumors, the biological functions of METTL1 vary considerably across different cancer types. While METTL1 exhibits clear oncogenic activity in certain malignancies, such as ICC and HCC (7, 43), where it promotes tumor proliferation, metastasis, and immune evasion, studies have also reported potential tumor-suppressive roles of METTL1 in other cancers, including breast cancer (41). Therefore, therapeutic strategies targeting METTL1 should be approached with caution. The concept of combining METTL1 inhibitors with ICIs may hold promise in specific contexts (e.g., ICC or HCC), but its efficacy is likely to depend on the cancer type, tumor microenvironment, and immune landscape. Future research should focus on elucidating the context-dependent functions of METTL1 and developing individualized therapeutic strategies based on tumor-specific molecular characteristics.

Despite these promising prospects, several challenges remain for the clinical application of METTL1-targeted therapies. First, as a widely expressed RNA-modifying enzyme involved in maintaining translational homeostasis in normal cells, global inhibition of METTL1 may cause toxicity and off-target effects, necessitating strategies for tumor-specific targeting. Second, there is currently a lack of highly efficient, selective, and clinically applicable small-molecule inhibitors of METTL1, posing substantial challenges for drug development. Third, tumor heterogeneity may lead to variable dependence on METTL1 inhibition across different cancer types, requiring systematic validation in large-scale clinical cohorts. Future research directions should focus on: (i) the development of highly selective small-molecule inhibitors or novel nucleic acid–based therapeutics; (ii) integrated analyses using transcriptomics, proteomics, and single-cell omics to dissect METTL1 function across distinct immune cell populations; and (iii) rational combination strategies to achieve precision and individualized cancer therapy.

## 6 Future directions and perspectives

Despite significant progress in recent years regarding METTL1 and its mediated m^7^G modification in cancer, numerous challenges remain. First, the regulatory mechanisms of METTL1 are not yet fully elucidated. Although existing studies suggest that its activity is modulated at multiple levels-including transcription factors, signaling pathways, and the cofactor WDR4-the precise molecular networks and dynamic regulatory patterns remain unclear. Additionally, validation in clinical samples and large-scale datasets is still limited, with most conclusions derived from *in vitro* studies or small patient cohorts. Large, multi-center, multi-omics studies are urgently needed to confirm the generalizability of METTL1’s roles across different cancer types and its association with clinical outcomes. Another critical challenge concerns substrate selectivity and specificity. While METTL1 is primarily recognized as a tRNA m^7^G methyltransferase, its preference for different tRNA species remains unclear, and it may even target other non-coding RNA molecules. Addressing this issue is essential not only for understanding the diverse cellular functions of METTL1 but also for guiding the design of highly specific therapeutic agents. Meanwhile, research on METTL1 role in tumor immune regulation remains relatively limited. Current evidence suggests that METTL1 may participate in immune evasion by influencing antigen processing and presentation and modulating immune cell function; however, the detailed mechanisms are still poorly defined. Further *in vivo* studies are needed to dissect its roles within the tumor immune microenvironment and to evaluate potential interactions with immune checkpoint pathways.

Emerging research directions offer promising avenues for future investigation. For example, experimental evidence indicates that METTL1 deficiency can enhance T cell–mediated antitumor responses, suggesting potential synergy with PD-1/PD-L1 blockade therapy. Future studies could explore strategies combining METTL1 genetic ablation or pharmacological inhibition with immunotherapy. Additionally, the relationship between RNA-modifying enzymes and liquid–liquid phase separation is gaining attention, raising the question of whether METTL1 regulates substrate selection and translational efficiency through phase-separated compartments. Animal models will be crucial for elucidating the causal roles of METTL1 in tumorigenesis, progression, and immune responses. Finally, therapeutic development remains a major challenge. Highly efficient and selective small-molecule inhibitors or nucleic acid–based drugs targeting METTL1 are currently lacking. Structure- and function-guided drug design, combined with rational combination strategies involving immunotherapy or targeted therapy, will be critical to translating METTL1-related research from the bench to the clinic.

## 7 Conclusion

In summary, METTL1, as a tRNA m^7^G methyltransferase, plays a multi-layered and critical role in cancer development and tumor immune regulation. By modulating tRNA stability, translational efficiency, and protein synthesis, METTL1 not only promotes tumor cell proliferation, migration, and metabolic reprogramming but may also contribute to immune evasion through its effects on antigen processing and immune cell function. Extensive studies have demonstrated that METTL1 is aberrantly expressed in multiple cancer types, with high expression closely associated with poor prognosis, highlighting its central role in cancer biology. Moreover, METTL1 exhibits significant potential for clinical translation. As a prospective biomarker, its expression levels could be utilized for tumor diagnosis, prognosis assessment, and prediction of immunotherapy response. Therapeutically, targeting METTL1 through small-molecule inhibitors, RNA interference strategies, or in combination with immune checkpoint blockade offers promising avenues for novel anticancer interventions. Despite current challenges, including substrate specificity, incomplete understanding of its immune regulatory mechanisms, and limited clinical validation, future research integrating multi-omics analyses, animal model studies, and targeted drug development may advance METTL1 from a basic research focus to clinical application, providing new strategies and opportunities for cancer therapy.
